# Joint Estimation of Time-Frequency Signature and DOA Based on STFD for Multicomponent Chirp Signals

**DOI:** 10.1155/2014/937139

**Published:** 2014-11-09

**Authors:** Ziyue Zhao, Congfeng Liu

**Affiliations:** Research Institute of Information Countermeasure, Xidian University, Xi'an, Shaanxi 710071, China

## Abstract

In the study of the joint estimation of time-frequency signature and direction of arrival (DOA) for multicomponent chirp signals, an estimation method based on spatial time-frequency distributions (STFDs) is proposed in this paper. Firstly, array signal model for multicomponent chirp signals is presented and then array processing is applied in time-frequency analysis to mitigate cross-terms. According to the results of the array processing, Hough transform is performed and the estimation of time-frequency signature is obtained. Subsequently, subspace method for DOA estimation based on STFD matrix is achieved. Simulation results demonstrate the validity of the proposed method.

## 1. Instruction

As a typical kind of nonstationary signal, chirp signal is widely used in the fields of radar, sonar, and communications. For the reconnaissance and measurement of this kind of signal, the prior information of time-frequency is always unknown. Consequently, an algorithm to estimate time-frequency signature and DOA jointly is necessary. Time-frequency analysis method has been applied in the field of the estimation of nonstationary signal's time-frequency parameters, and it acquires a great deal of achievements [1, 2]. The pseudo Wigner-Ville distribution (PWVD) is one of the most commonly used time-frequency analysis tools, which can gain quite high resolution in both time domain and frequency domain and mitigate cross-terms in certain degree for multiple chirp signals [3, 4]. On the other hand, with the combination of time-frequency distributions (TFDs) and array signal processing, the performance of algorithm for spatial processing will be improved since the information of time-frequency is also considered. In the meanwhile, array processing also contributes to the mitigation of cross-terms of TFDs. Hereafter, the joint estimation of time-frequency signature and DOA is achieved.

In order to obtain the directions of arrivals (DOAs) estimation based on TFDs, the STFD is proposed by Amin et al. [5, 6]. This model is presented in the condition of narrowband nonstationary signals where the variation of signal frequency is much smaller than carrier frequency. In order to apply this model to wideband nonstationary signal, Gershman and Amin [7] constructed the corresponding array signal model. Array signal processing and TFDs are combined in this model and the conventional data covariance matrix is replaced by STFD matrix based on time-frequency points (*t-f* points) in subspace estimation methods. On the one hand, as a result of the utilization of the spatial and time-frequency information, the STFD-based DOA estimation method improves angular resolution performance and is more robust than conventional subspace estimation method [8]. On the other hand, the cross-terms will be suppressed to a great extent when performing array processing for TFDs, which offers effective support for the estimation of time-frequency signature and the selection of* t-f* points.

In this paper, the STFD model is applied to estimate time-frequency signature and DOA jointly, where the method for DOAs estimation of multicomponent chirp signals based on multiple* t-f* points is proposed and array processing is applied to the estimation of time-frequency signature. Firstly, the signal model is presented and nonstationary environments defined by chirp signals are considered. Then, the estimation of time-frequency signature based on array processing is performed and the instantaneous frequencies of chirp signals are achieved. Meanwhile, STFD matrices based on multiple* t-f* points are constructed. Finally, the DOAs estimation via searching the top values of the sum function of spatial spectrum is obtained. The results of the simulation demonstrate the validity of the method for multicomponent chirp signals.

## 2. Signal Model

Consider *P* wideband chirp signals impinging on a uniform linear array (ULA) consisting of *M*  (*M* > *P*) sensors. So the received signal vector can be expressed as

(1) $$\begin{array}{c} x\left(t\right)=A\left(\theta ,t\right)s\left(t\right)+n\left(t\right), \end{array}$$

where **x**(*t*) = [*x*_1_(*t*), *x*_2_(*t*),…,*x*_*M*_(*t*)]^*T*^ is the *M* × 1 received signal vector, **A**(***θ***, *t*) = [**a**(*θ*_1_, *t*), **a**(*θ*_2_, *t*),…, **a**(*θ*_*P*_, *t*)] is the *M* × *P* direction matrix, **a**(*θ*_*i*_, *t*) is the *M* × 1 time-varying direction vector of the *i*th signal at the time *t*, ***θ*** = [*θ*_1_, *θ*_2_,…, *θ*_*P*_] is the direction vectors of *P* signals, **s**(*t*) = [*s*_1_(*t*), *s*_2_(*t*),…,*s*_*P*_(*t*)]^*T*^ is the *P* × 1 vector of signal waveforms at the time *t*, **n**(*t*) = [*n*_1_(*t*), *n*_2_(*t*),…, *n*_*M*_(*t*)]^*T*^ is the *M* × 1 vector of additive white Gaussian noise with variance of *σ* ^2^, and ()^*T*^ denotes transpose. As the nonstationary characteristic of the signal frequency, the time-varying direction vector is

(2) aθi,t=1,exp⁡−j2πfitcdsinθi,…,hhhexp⁡−j2πfitcM−1dsinθiT,

where *f*_*i*_(*t*) is the instantaneous frequency of the *i*th signal, *c* is the speed of light, and *d* is the array interelement spacing, which meets the requirement of half wavelength.

## 3. Time-Frequency Signature and DOA Joint Estimation

### 3.1. Construction of STFD Matrix

In order to build the STFD matrix, we first give the discrete form of PWVD of the signal *x*(*t*):

(3) $$\begin{array}{c} D_{xx}\left(t,f\right)=\overset{(L-1)/2}{\underset{l=-(L-1)/2}{\sum }}x\left(t+l\right)x^{*}\left(t-l\right)e^{-j4\pi fl}, \end{array}$$

where *t* and *f* represent the time and frequency indexes, respectively, *L* is the length of the window function, and ()^*^ denotes complex conjugate. Then, substituting (1) into (3) and taking the expectation, we obtain the STFD matrix [9]

(4) Dxxt,f=E∑l=−(L−1)/2(L−1)/2x(t+l)xH(t−l)e−j4πfl=∑l=−(L−1)/2(L−1)/2Aθ,t+lD^sst,f,lAHθ,t−l+σ2I,

where ${\hat{D}}_{ss}(t,f,l)=E\left[s(t+l)s^{H}(t-l)e^{-j4\pi fl}\right]$ and ()^*H*^ denotes conjugate transpose.

We can see that the direction matrix is still changing in the window length because of the time-varying of the instantaneous frequency. For the purpose of simplifying the STFD matrix (4) and applying the subspace methods to find DOA, the window length *L* can be restricted by [7]

(5) a(θi,t1)−a(θi,t2)  ≪MP, ∀t1,t2∈t−L−12,t+L−12.

Then, (4) can be approximately written as

(6) $$\begin{array}{c} D_{xx}\left(t,f\right)\approx A\left(\theta ,t\right)D_{ss}\left(t,f\right)A^{H}\left(\theta ,t\right)+\sigma^{2}I, \end{array}$$

where (**D**_*xx*_(*t*, *f*))_*ij*_ = **D**_*x*_*i*_*x*_*j*__(*t*, *f*), *i*, *j* = 1,2,…, *n* and $D_{ss}(t,f)=\sum_{l=-(L-1)/2}^{(L-1)/2}{\hat{D}}_{ss}(t,f,l)$ is the TFDs matrix of **s**(*t*), which consists of autosource TFDs as the diagonal elements and cross-source TFDs as the off-diagonal elements. The STFD matrix and the source TFDs matrix in (6) are similar to the spatial covariance matrix and the source covariance matrix. So it is clear that the two subspaces spanned by the principle eigenvectors of **D**_*xx*_(*t*, *f*) and the columns of **A**(***θ***, *t*) are identical so that the subspace method can be used here.

As it can be seen from (6), the construction of the STFD matrix from the* t-f* points of highly localized signal energy allows the enhancement of the signal-to-noise ratio (SNR), which is of great significance to the performance improvement of DOA estimation [10]. Therefore, in order to choose the appropriate* t-f* points, it is necessary to mitigate cross-terms in TFDs and obtain the instantaneous frequency of signal. In the meanwhile, for the accurate estimation of the signal time-frequency signature, TFDs should also be processed. Subsequently, the method to reduce cross-terms contamination and to enhance the true signal* t-f* power concentration will be discussed.

### 3.2. Estimation of Time-Frequency Signature

On the condition that the receiver is a single sensor, PWVD has always been utilized to reduce the cross-terms of Wigner-Ville distribution (WVD). It is a mature method in reduction of cross-terms, which smoothes the WVD by a rectangular window since the cross-terms are oscillating. However, when the receiver is array antenna, the spatial information received from it can be utilized to reduce the cross-terms, and the simulation results will show that this processing method can achieve better performance compared with PWVD in reduction of cross-terms.

Firstly, as a basic method of array processing, array averaging of WVD can be expressed as follows, and the noise is ignored in the following deduction [11]:

(7) W−t,f=1M∑m=1MWxmxmt,f=∑i=1P∑j=1PβijWsisjt,f,

where **W**_*x*_*m*_*x*_*m*__(*t*, *f*) is WVD of the *m*th sensor and **W**_*s*_*i*_*s*_*j*__(*t*, *f*) is the cross-WVD between the *i*th and *j*th signal. **W**_*s*_*i*_*s*_*j*__(*t*, *f*) corresponds to autoterms or cross-terms of WVD, depending on whether *i* = *j* or *i* ≠ *j*. Spatial correlation coefficient *β*_*ij*_ = (**a**_*j*_^*H*^ **a**_*i*_)/*M* has the feature that

(8) $$\begin{array}{c} \beta_{ij}\left\{\begin{array}{cc} \leq 1, & i\neq j,\\ =1, & i=j. \end{array}\right. \end{array}$$

It can be seen that, as the weight of autoterms and cross-terms, the spatial correlation coefficient *β*_*ij*_ for autoterms is always greater than, or at least equal to, those for the cross-terms, which means that array averaging of WVD can suppress cross-terms in a certain degree.

For further suppression of cross-terms, we hope the weight to be ones and zeros for autoterms and cross-terms, respectively, that is, impulse function. In this way, the autoterms are maintained and the cross-terms are entirely eliminated. To this end, the received signal should be prewhitened and then array averaging in beamspace should be performed [12, 13].

The covariance matrix can be written as

(9) Rxx=Ex(t)xH(t)=ARssAH+σ2I,

where **R**_*ss*_ is the diagonal matrix with signals' variance when the signals are pairwise independence. Then, perform eigenvalue decomposition for **R**_*xx*_ and sort the eigenvalue as *λ*_1_ > *λ*_2_ > ⋯*λ*_*P*_ > *λ*_*P*+1_ = ⋯*λ*_*M*_ = *σ* ^2^. Utilize the top *p* eigenvalue to constitute diagonal matrix Λ_*s*_ and utilize the corresponding eigenvector to make up matrix **U**_*s*_. Hereafter, the prewhitening matrix can be expressed as

(10) $$\begin{array}{c} T^{H}={\left(\Lambda_{s}-\sigma^{2}I_{P}\right)}^{-1/2}U_{s}^{H}. \end{array}$$

This prewhitening matrix can be used as beamspace transformation matrix, and the array averaging in beamspace can be written as

(11) W−bst,f=1P∑p=1PWypypt,f=∑i=1P∑j=1PδijWsisjt,f,

where **W**_*y*_*p*_*y*_*p*__(*t*, *f*) is the WVD of the *p*th beam and the output of array is **y**(*t*) = **T** ^*H*^ **x**(*t*) and *δ*_*ij*_ is impulse function. It can be expressed as

(12) $$\begin{array}{c} \delta_{ij}=\left\{\begin{array}{cc} 0, & i\neq j\\ 1, & i=j. \end{array}\right. \end{array}$$

It is clear that the array averaged WVD in beamspace can suppress the cross-terms more thoroughly when the two weight functions are compared. Consequently, the latter method is more appropriate for estimation of time-frequency signature and the selection of* t-f* points.

In order to estimate the time-frequency signature of every component of chirp signal, the initial frequency and chirp rate, the Hough transform can be used here to detect the lines in time-frequency plane (*t-f* plane) and then to calculate the parameters. The Hough transform can convert a straight line in image space into a peak in a parameter space. The Hough transform can be expressed as *ρ* = *x*cos⁡*θ* + *y*sin*θ*, where (*x*, *y*) is the coordinate of a point in image space and *ρ* is the normal distance from the origin to the line and *θ* is the angle the normal line makes with *x*-axis. Accordingly, points from the same line in the image intersect at one point in parameter space and accumulate as a peak after the transform of all the points in the image [14].

Hence, performing the Hough transform in* t-f* plane can produce peaks for the chirp signals since the signals are represented as lines in WVD. At the same time, because the cross-terms in proposed WVD are suppressed in a great degree, the lines of cross-terms in* t-f* plane will not form considerable high peaks after the Hough transform, which is more easily used to detect the peaks of real signals.

**Figure 1:**
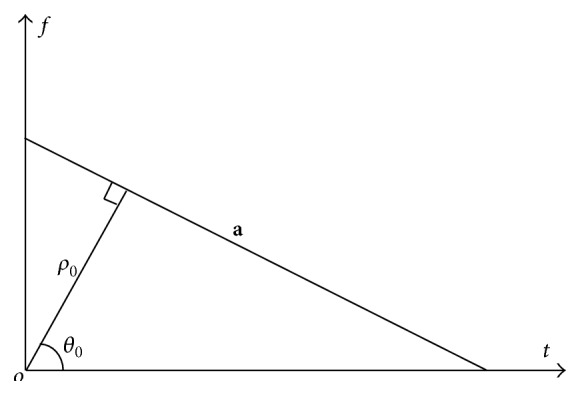
Hough transform schematic.

Then, according to the coordinate of the peak in parameter space, the initial frequency and chirp rate of chirp signals can be worked out. As shown in Figure 1, the line ***a*** represents a chirp signal in* t-f* plane and (*ρ*_0_, *θ*_0_) is the peak coordinate. Since the initial frequency and chirp rate are equal to the intercept and the slope of line ***a***, according to the geometrical relationship, they can be calculated as follows:

(13) $$\begin{array}{c} {\hat{f}}_{0}=\frac{\rho_{0}}{\sin\theta_{0}},\\ \hat{g}=-\text{cot}\;\theta_{0}, \end{array}$$

where ${\hat{f}}_{0}$ and $\hat{g}$ are the estimation value of two time-frequency parameters.

### 3.3. DOA Estimation Based on STFD

The STFD matrix **D**_*xx*_(*t*, *f*_*p*_(*t*)) based on one* t-f* point can be obtained from a component of chirp signals. Then, perform eigenvalue composition for the matrix and span signal subspace **U**_*s*_ and noise subspace **U**_*n*_ by using eigenvector of the maximum eigenvalue and other eigenvectors, respectively [15]. Because **U**_*s*_ and **U**_*n*_ are orthogonal and **U**_*s*_ is in the same subspace with direction matrix, **U**_*s*_ and the **U**_*s*_ subspace spanned by the columns of direction matrix are also orthogonal [16]. Finally, search the maximum value of the spatial spectrum function (**a** ^*H*^(*θ*_*p*_, *t*)**U**_*n*_ **U**_*n*_^*H*^ **a**(*θ*_*p*_, *t*))^−1^ and obtain the DOA estimation.

It is not robust enough for DOA estimation to use only one* t-f* point, and there will be relatively large error if the selected* t-f* point is not in the true instantaneous frequency region of* t-f* plane. Also, the DOAs of multiple components cannot be estimated at one time. In order to modify this, a novel method has been proposed to estimate DOAs for multicomponent chirp signals based on multiple* t-f* points.

Firstly, choose *P* components of the chirp signals and select *N t-f* points from every selected component and establish the STFD matrix of every* t-f* point. Then, perform matrix composition for these matrices and make up spatial spectrum functions. Subsequently, synthesize all selected* t-f* points to construct sum function of spatial spectrum

(14) $$\begin{array}{c} P_{\Sigma }=\overset{P}{\underset{p=1}{\sum }}\overset{N}{\underset{l=1}{\sum }}\frac{1}{a^{H}\left(\theta_{p},t_{l}\right)U_{n}\left(p,t_{l}\right)U_{n}^{H}\left(p,t_{l}\right)a\left(\theta_{p},t_{l}\right)}, \end{array}$$

where **a**(*θ*_*p*_, *t*_*l*_) and **U**_*n*_(*p*, *t*_*l*_) are direction vector and noise subspace of the *p*th signal in time *t*_*l*_. Ultimately, searching top *P* values of this function and the corresponding angles are the estimation values of DOAs.

On one hand, this method of DOA estimation is able to work in low SNR due to the preprocess in* t-f* plane. On the other hand, since the searching function of this method is constructed by multiple* t-f* points, the estimation values of DOAs can be obtained at one time and the proposed method is more robust than one* t-f* point based method [17].

## 4. Simulation Results

We assume a ULA of eight sensors spaced by half a wavelength and three chirp signals incident on the array. The observation period corresponds to 1024 samples.These chirp signals can be presented as *s*_*i*_(*t*) = *A*_*i*_ *e* ^*j*2*π*(*f*_*i*_*t* + *g*_*i*_*t*^2^/2)^, *i* = 1,2, 3 and the parameters of the chirp signals are set in Table 1. The noise here is additive Gaussian white noise and the SNR of the *i*th signal is defined as SNR_*i*_ = 10log⁡(*A*_*i*_^2^/*σ* ^2^), where *A*_*i*_ is the amplitude of the *i*th signals and *σ* ^2^ is the variance of noise.

**Figure 2:**
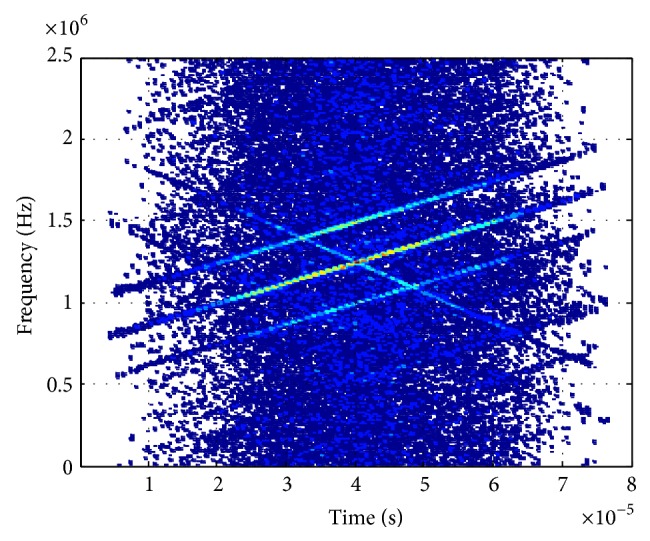
WVD (reference sensor).

**Figure 3:**
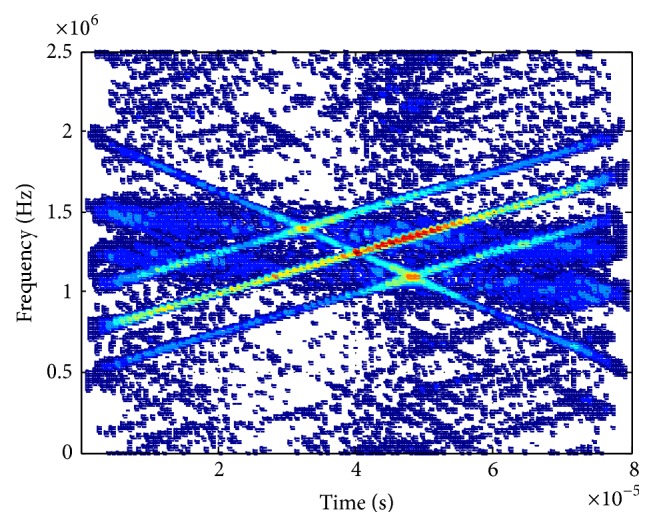
PWVD (reference sensor).

**Figure 4:**
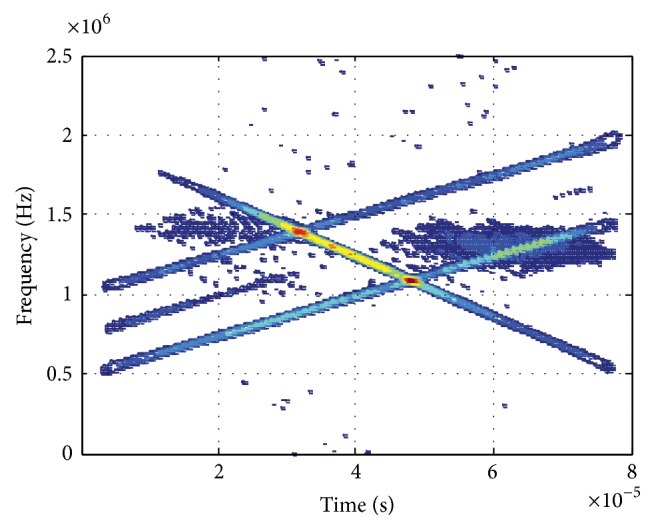
WVD (array averaged in beamspace).

Figure 2 displays the WVD of signals received by reference sensor where we can see the cross-terms are quite intense and it is difficult to find out the real chirp signals. Figures 3 and 4 show the PWVD of signals received by reference sensor with *L* = 256 samples and the array averaged WVD in beamspace, respectively, which are both the methods utilized to reduce the cross-terms of WVD. However, we can see the suppression of cross-terms is more efficient in Figure 4 compared with Figure 3.

**Figure 5:**
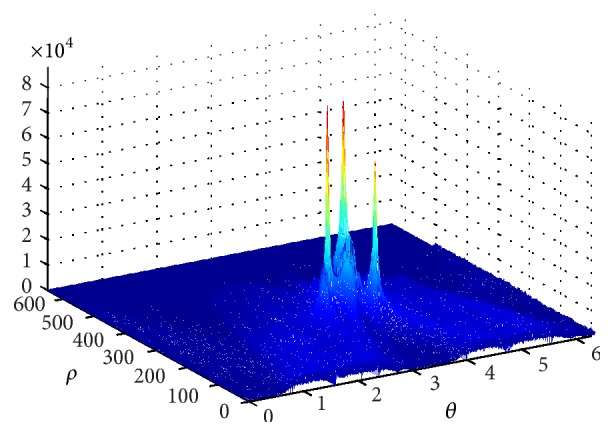
Hough transform result.

Then, the result of Hough transform of Figure 4 is displayed in Figure 5. After that, inserting the coordinate of the peaks into (13), we obtain the estimation values of initial frequencies which are ${\hat{f}}_{1}=2.0120$ MHz, ${\hat{f}}_{2}=0.4875$ MHz, and ${\hat{f}}_{2}=0.9874$ MHz. Similarly, the estimation values of chirp rates are ${\hat{g}}_{1}=-1.9344\times 10^{4}$ MHz/s, ${\hat{g}}_{2}=1.2838\times 10^{4}$ MHz/s, and ${\hat{g}}_{3}=1.2838\times 10^{4}$ MHz/s.

**Figure 6:**
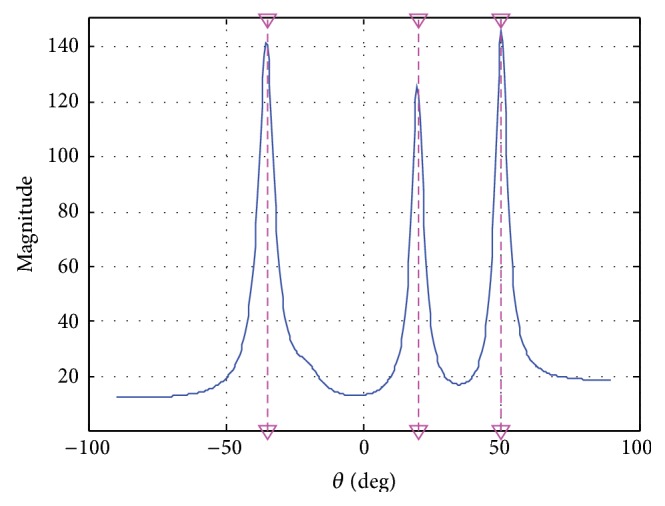
Spatial spectra.

Finally, choose the* t-f* points of each signal component in the vicinity of time midpoint where the signal* t-f* signature is quite clear and calculate the sum function of spatial spectrum based on (14) with *P* = 3 and *N* = 32.  Figure 6 shows the calculated spatial spectrum and according to the peaks in the figure the reasonable estimation values of DOAs are achieved.

**Figure 7:**
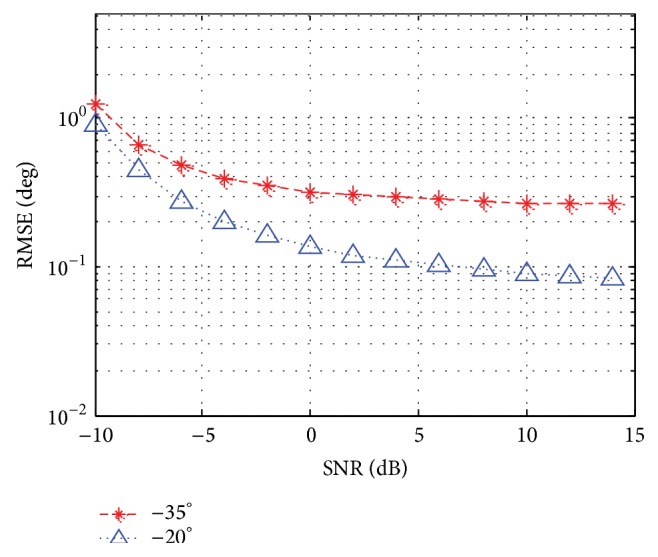
RMSE versus SNR.

**Figure 8:**
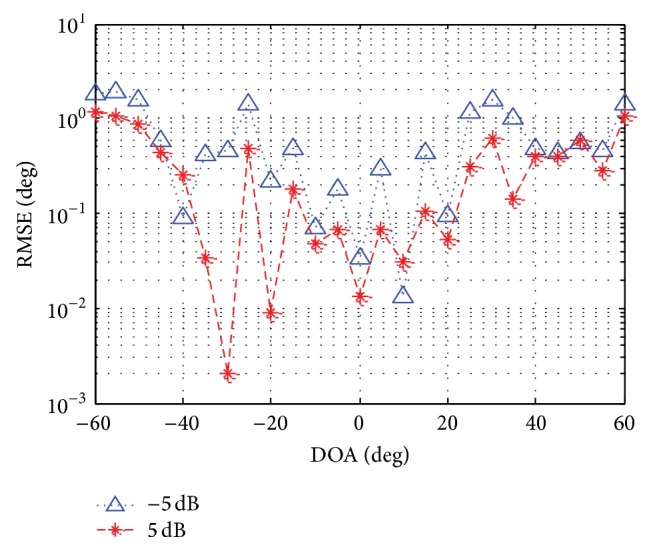
RMSE versus DOA.

In the following simulation, we analyze the performance of DOA estimation.  Figure 7 displays the root mean square error (RMSE) of the estimated DOA versus SNR for the case (*θ*_1_, *θ*_2_) = (−35°, −20°). From this figure it can be seen that the proposed method also works when the SNR is quite low.  Figure 8 displays the curves of the RMSE versus DOA and in this case SNR is 5 dB and −5 dB, respectively. It is clear that the estimation performances are similar in symmetrical azimuths and the RMSE increases with the augment of DOA.

## 5. Conclusions

In this paper, the method applied to estimate time-frequency signature and DOA jointly for multiple components of chirp signal based on the spatial time-frequency distribution model is proposed. By applying array averaging in beamspace, the cross-terms of TFDs are suppressed to a significant degree, which lays solid foundation for estimation of time-frequency signature precisely. In the meanwhile, the proposed method of DOA estimation based on sum function of spatial spectrum can obtain DOA results precisely even in unsatisfactory conditions. The simulation results show that the proposed method for joint estimation of time-frequency signature and DOA is valid.

## Figures and Tables

**Table 1:** Simulation parameter setting.

	Initial frequency	Chirp rate	DOA	SNR
Signal 1	*f*_1_ = 2 MHz	*g*1 = −1.875 MHz/s	−35°	0 dB
Signal 2	*f*_2_ = 1 MHz	*g*2 = 1.25 MHz/s	50°	0 dB
Signal 3	*f*_3_ = 0.5 MHz	*g*3 = 1.25 MHz/s	20°	0 dB
